# Moving Hong Kong towards a tobacco-free generation: rebutting counter arguments

**DOI:** 10.1016/j.lanwpc.2024.101064

**Published:** 2024-04-08

**Authors:** Aaron H.L. Wong

**Affiliations:** Department of Applied Social Sciences, The Hong Kong Polytechnic University, Hong Kong, China

Tobacco causes over 6900 deaths in Hong Kong and 7 million deaths worldwide every year.^1^ Aiming at moving towards “a vibrant, healthy and tobacco-free Hong Kong”, the Hong Kong government has consulted the public on, and is currently considering the proposal of enacting a tobacco-free generation law. The law, if enacted, will introduce a lifetime tobacco sales ban on the younger generations, who are born after a specified date and currently below the legal smoking age of 18.^2^ To gradually achieve tobacco endgame, the UK government is also proposing the enactment of such a law, despite New Zealand's recent decision to repeal its tobacco-free generation law.^3^

This comment evaluates and rebuts four objections, raised not only in Hong Kong but also in other jurisdictions, against the enactment of a tobacco-free generation law, that it (1) makes smoking appear tempting; (2) fuels the growth of the black market; (3) infringes personal freedom; and (4) is discriminatory.^4^^,^^5^

First, it is unlikely that illegalisation would make tobacco products more tempting. Since 1982, Hong Kong has introduced a wide range of tobacco control measures, including the prohibition of tobacco sales to persons under 18 years old, printing mandatory health warnings on tobacco products, and a full smoking ban on indoor public places.^1^ With 40 years of tobacco control efforts, especially in promoting the negative health effects of smoking, Hong Kong successfully lowered its smoking prevalence from 23.3% in 1982^1^ to 9.5% in 2021.^2^ The current smoking prevalence of secondary 1–6 students, who are under the legal age to purchase tobacco products, is around 1.2%.^2^ A lifetime tobacco sales ban targeted at the same group, most of whom either view smoking unfavourably or are deterred by the law from smoking, would likely only reinforce the perceived negativity of smoking.^6^

Second, the tobacco-free generation law would not fuel the growth of the black market. Existing smokers need not turn to the black market since they are not affected by the proposed law.^7^ The current underage smoking prevalence of 1.2% hardly provides sufficient demand for a large tobacco black market to grow, not least to a scale comparable to the current white market. As most of those who will be subjected to the tobacco-free generation law are not addicted to tobacco, removing the social legitimacy of smoking^7^ and the threat of criminal sanctions will strongly discourage them from starting to smoke. Recently, research also found that prohibitions on sales and use are generally effective in reducing smokeless tobacco use.^8^ Furthermore, Hong Kong's strong enforcement and punitive sentencing would likely deter illegal sales and use of tobacco when the generation ban is in force. As a reference, Hong Kong's tough approach towards illicit drugs has kept their use at a low level, with the number of reported drug abusers only accounting for 0.08% of the total population in 2021.^9^ Therefore, the tobacco-free generation law would likely reduce the overall demand and supply of tobacco products.

Two normative objections remain. It is said that a generation ban infringes personal autonomy and liberty by prohibiting (later grown) adults from making an informed choice to smoke. This line of argument suggests that criminal law should be reserved to govern the most harmful activities, and many socially undesirable activities, such as gambling and binge drinking, are not always prohibited by the law. However, the justification for banning tobacco use is sufficiently strong. The enormous number of tobacco-related deaths and the huge public health expenditures for treating tobacco-related illnesses constitute grave social harm. Moreover, the high addictiveness of tobacco undermines people's autonomy to quit smoking. Decisions to start smoking were often not a fully informed choice,^10^ and over 90% of adult smokers regretted having started smoking.^11^ Secondhand smoke also jeopardises the health of others who did not make a voluntary choice to smoke.^12^ With all these harms, a lifetime ban on tobacco can be justified for protecting citizens' right to life and right to health.^12^

The final objection is that a generation ban unfairly discriminates between different age groups as it allows some to purchase tobacco while prohibiting the younger generation from doing the same. In response, the differentiation can be fully justified by the legal proportionality test. Imposing an absolute ban on those who were already addicted to tobacco may impose an unacceptably harsh burden on them, and is therefore disproportionate.^13^ In contrast, imposing a lifetime ban only on those who have never smoked strikes a reasonable balance by concurrently reducing the harm caused by tobacco use and minimising the level of distress caused to citizens.

## Declaration of interests

None.
